# Association of information, education, and communication with enrolment in health insurance: a case of Nepal

**DOI:** 10.1186/s13690-020-00518-8

**Published:** 2020-12-14

**Authors:** Devaraj Acharya, Bhimsen Devkota, Kamal Gautam, Radha Bhattarai

**Affiliations:** 1 Bhairahawa Multiple Campus, Tribhuvan University, Siddharthanagar, Rupandehi Nepal; 2 Mahendra Ratna Campus, TU, Kathmandu, Nepal; 3 Central Department of Education, TU, Kirtipur, Kathmandu Nepal; 4 Bhairahawa Multiple Campus, TU, Bhairahawa, Nepal

**Keywords:** Enrolment, Health insurance, Information, education, and communication, Nepal

## Abstract

**Background:**

Many studies indicate that various health programmes have been failed because of the lack of appropriate information, education, and communication [IEC] for the target audiences. It is still unanswered which methods/means of communication could be the most powerful for changing behaviour or decision-making capacity. The paper aims to assess the effects of IEC on family enrolment in health insurance programme [HIP] in Nepal.

**Methods:**

We employed a household-based observational study with a control group. Altogether 810 household interviews were conducted in Baglung and Kailali districts of Nepal in 2018. The study used a validated structured interview schedule. Background characteristics of the family and respondents and their exposure to the means of communication were the independent variables while enrolment in health insurance [HI] was the dependent variable.

**Results:**

Data showed that 72% of the respondents heard about the HI and 66% knew the contribution amount for enrolment in HI. In the total enrolled households, 53% were household heads, 59% belonged to the age group 41–60 and 68% were above 60 years. More than half (56%) of rich compared to 46 and 49% of middle and poor (*p* < 0.05); 60% of the family member suffering from the chronic disease were enrolled in the HI. Similarly, 68% of those who heard about HI compared to 4 % who did not hear were enrolled (*p* < 0.001). A vast majority (69%) of those knowing contribution amount, 73% who interact with peer neighbour compared to 39% who did not, and 62% of those who listened to the radio and 63% of those who watched TV were enrolled in HI (*p* < 0.001). However, heard about HI (aOR = 21.18, 95%CI: 10.17–44.13, *p* < 0.001), knowledge about contribution amount (aOR = 5.13, 95%CI: 3.09–8.52, *p* < 0.001), having HI related books or guidelines (aOR = 4.84, 95%CI: 2.61–8.98, *p* < 0.001), and interact with peer or neighbours (aOR = 1.74, 95%CI: 1.34–2.65, *p* < 0.01) were appeared to be positive and significant predictors for enrolment in HI.

**Conclusion:**

Knowledge about HI and interaction with peers and neighbours about the HI scheme of the government could lead to higher participation in the HIP. It would be better to incorporate this strategy while planning interventions for increasing enrolment in the HIP.

**Supplementary Information:**

The online version contains supplementary material available at 10.1186/s13690-020-00518-8.

## Background

Government of Nepal [GoN] has committed to achieve Universal Health Coverage [UHC] by 2030 which is one of the targets of Sustainable Development Goals [SDGs] for good health and wellbeing [SDG 3] [1]. Besides this, the Constitution of Nepal [CoN] has also declared basic health services as one of the fundamental rights of the citizens [2]. However, the GoN has allocated less than 3 % of its total budget for the health sector [3] which is said to be insufficient to meet the targets of the global agenda of good health and wellbeing for all and the constitutional provision of the right to healthcare. Therefore, appropriate and sustainable financing for health is needed to meet the targets and agendas. The GoN has formulated the Health Insurance Act [HIA] 2017 to ascertain the financial sustainability for health care [3, 4]. Health Insurance [HI] programme was initially introduced as Social Health Security [SHS] in 2016 under the provision of the Development Board Act 1956 in Kailali, Baglung, and Ilam Districts in the initial phase [5].

The Health Insurance Programme [HIP] is relatively a new programme for Nepalese people. So, it has both opportunities and challenges to implement. The opportunity in the sense of a new programme to the households and the challenge in the sense that people may or may not participate in the HIP since they may not have adequate and correct information about it. A survey conducted in Kailali District shows that only 9 % of people had good knowledge about HI [6]. The HIP started in May and August 2016 in Kailali and Baglung Districts respectively in the initial phase [7]. Before 2019, a five-member family had to pay Nepalese Rupee (NRs.) 2500 (US$ 23.89 as of 15th April 2018) per year for enrolment, and an additional member needed to pay NRs 425 (US$ 4.06) each for enrolment. However, the amount has been increased to NRs. 3500 (US$ 33.45) at present. During data collection, the coverage amount was NRs. 50,000 (US$ 477.87), however, it is now increased to NRs.100000 (US$ 955.75) with a maximum ceiling of NRs. 200,000 [7]. By the end of the fiscal year 2017/2018, a total of 42 districts out of 77 districts were covered by the HIP. Only 8 % of the population were enrolled in the HIP as of November 2018 from 36 districts but less than three [2.4] percent of the population were enrolled in Baglung and Kailali Districts, and the majority did not renew their scheme [3, 5]. People want to enrol and pay more than the contribution amount if quality services available to them but enrolment rate appeared low [8]. This may have happened because of inadequate information, education, and communication [IEC] activities. Inadequate IEC leads to poor enrolment, lower retention, and poor renewal rate as well.

The HIP in the context of Nepal requires proper sensitization and information to the targeted population at the mass level. Various interventions such as sensitization, awareness, orientation, and training shall be conducted for mass enrolment [9, 10]. However, it is still unanswered which method would be more appropriate to get people informed about HIP. The Health Insurance Board [HIB] has set three tiers of communication strategy at the policy level, community level, and household level but the strategy is yet to be validated [11]. IEC is a combination of strategies, methods, and approaches that enables a person to adopt a dynamic role in improving quality of life through healthy conduct [12]. IEC is not only limited to the process of changing behaviour but also a process of political, social, and economic transformation. Adequate IEC approaches can encourage and support to follow up for positive behaviour change [13]. IEC creates awareness, increases knowledge, changes attitude, and moves people towards change and continues their behaviours to adopt an innovation [14]. It updates and upgrades knowledge, awareness, and attitudes for a favourable change in behaviour or decision making [15, 16].

Nepal Demographic and Health Survey [NDHS] 2016 shows that more than half [50% of women and 51% of men] of young adults [15–49 years] had access to television [TV]. They watched TV at least once a week, consequently 27.7% of women and 36.1% of men had access to radio and listened at least once a week, and 37.2% of women and 31% of men had no access to newspapers, TV, or radio at least once a week. However, 24% of women and 50% of men had access to the internet and they used it within the past 12 months [17]. Nearly half of the population had no access to mass media which may prevent them from accessing health-related information including health insurance. NDHS 2016 further indicates that TV was the most common media and half of the people had access to it [17].

Enrolment in HI might be observed from different perspective such as legal, economic, social, and developmental. This study observed the HI programme from the behaviour change perspective. Good and healthy behaviours are often time-consuming, costly, difficult, inconvenient, complicated, and even less rewarded. Therefore, people generally do not follow healthy behaviour [18]. Rather it leads to a negative attitude towards healthy behaviours. So, it needs appropriate intervention to overcome the negative attitude. A bad IEC could damage wellbeing of individuals but a good IEC could lead to change their behaviour and lives positively [19]. Gathering all people in the mainstream of IEC is a difficult task. It does not only change the behaviour but develops culture and civilization. It is a process of transforming innovations, ideas, opinions, and new trends [20].

IEC informs, inspires, motivates, enables, and empowers people for deciding the healthy way by making changes in terms of knowledge, attitudes, and beliefs [21]. Communication is the power for decision making and behaviour change. It makes individuals positive, motivating, encouraging, and supportive for understanding [22]. IEC consists of several methods, approaches, and interventions but it is neither evaluated nor assessed which method and approach would be better for behaviour change concerning enrolment in health insurance in the context of Nepal. So, the article aims to assess the association of information, education, and communication on enrolment in health insurance.

## Methods

### Research design

A household-based observational study with control group was used. The control group was composed of households which had not enrolled in the health insurance programme at the time of the study.

### Study setting and period

The research sites were Baglung and Kailali Districts of Nepal. Baglung is located in the hilly region in the mid-western part and Kailali is situated in Terai in the south-western part of Nepal. In these districts, HIP was implemented in the initial phase [3]. We chose Baglung form Hill and Kailali from Terai, the southern plain. Data collection took nearly 11 weeks to complete.

### Study participants

Enrolled and non-enrolled household heads [HH] were the respondents of the study. In case of absence or rejection to respond by the HH, another senior member of the family was requested to respond. The assumption of collecting information from the HHs was that they may have more information about family and family-related information compared to other members of the family.

### Variables

Socio-demographic characteristics of respondents and households such as age, sex, household headship (who were involved in decision making of the household such as purchasing of goods, health-related decisions), health status (a household with a family member suffering from chronic disease(s) and taking medicine in a regular basis such as high blood pressure, kidney diseases, diabetes), exposure to communication and media (radio, TV, newspaper, hoarding board, and poster pamphlets, etc.) were independent variables and enrolment in HI was the dependent variable. Different nine types of household assets and dwelling were assessed to categorize wealth status in three equal classes [17]. So, the wealth status of the people comprised one third each of the rich, middle, and poor households.

In the study, the enrolled households are those registered in the Governmental Health Insurance Programme, a government body named Health Insurance Board [HIB] (before 2017 it was named as Social Health Security Development Committee). According to the provision, the HIP covers up to five members of the family for a flat NRs. 2500/− and additional members have to pay an amount of NR 425 each. However, the amount has been changed to NRs. 3500 for up to five-member family. The household that did not enrol in the HIP of the HIB is considered as non-enrolled household.

Households enrolled in the HIP before 15th January 2018 from the Health Insurance Board [government funded body] were included [for enrolled sample] in the study. Individuals or families enrolled from private or other insurance companies or any other welfare programmes [such as welfare/medical scheme for Nepalese/Indian/ British Ex-army] were excluded in the listing of the enrolled households in the initial phase. Therefore, these HHs were automatically excluded and were not included in the analysis. In the case of non-enrolled samples, proximal households in terms of distance from the enrolled households were included for non-enrolled sample. In case of having more than one household in the proximity, simple random sampling was applied to select the non-enrolled household.

### Population and sample size

All the households residing in Baglung and Kailali Districts were the population of the study. There were two types of samples: enrolled and non-enrolled households. The required sample size was calculated by using online Survey Monkey software [23] among the population determined by the latest National Census and Households Survey 2011. There were 204,002 households [61,522 and 142,480 households in Baglung and Kailali respectively] [24] with a confidence level of 95% and a 5 % margin of error. It showed an estimated sample size of 384.2 ~ 385 [25, 26].

By adjusting the non-response rate of 5 % as evidenced by NDHS 2016 [17], the sample became 405 for enrolled families and the same sample size was determined for the non-enrolled households. Sample for enrolled households accounted for 122 for Baglung and 283 for Kailali as per population proportion to size [24] and the same for non-enrolled households. The list of households was obtained from HIB district offices. The household unit was randomly selected for the enrolled sample. There were 9779 households enrolled in the HI programme by mid-January, 2018 according to the record of the Health Insurance Board, District Offices. The proximal (adjoining: in terms of distance) household of an enrolled household, which was not enrolled in the HIP, was selected as a non-enrolled sample, random sampling was used if more than one household in the proximity.

### Data collection tool

Interview schedule [IS] was used for data collection. Five percent of the total sample (*n* = 82) was pre-tested and modified for the validation of the study tool before administration. The IS was validated by the test-retest method which had eight sections. IS was pre-tested and amended four times inside and outside the study area. Cronbach’s alpha was calculated for the validation of attitude statements which accounted for 0.734 since the score of more than 0.70 is acceptable to administer [27]. But only the results of the IEC related section have been presented in this article. As an additional file, the IS attached to the article.

### Data collection procedure

After completing all administrative procedures: ethical approval was received from Nepal Health Research Council [NHRC], obtained permission from HIB central and district offices, we intended to collect data from the household heads [HHs]. Informed consent was taken before interviewing. Data were collected from respondents’ houses or the place where they felt comfortable for an interview or response. A two-day training was provided to the enumerators about research ethics, techniques for data collection, and other research-related topics.

The study yielded an almost 100% response rate. However, 2.47% [20] of the total respondents [HHs] refused (hesitated) to respond indicating that another member of the family had more knowledge about HI compared to them. Therefore, data were collected from another senior member of the household in such instance. Almost all data as per the interview schedule were collected from the respondents. Data collection was started on 20th March and completed on 5th June 2018.

### Data quality management

The study used a validated and pre-tested interview schedule. There were four levels of data quality management strategies. First, the spot check was done right after completing the interview. Second, 20% (162 samples) of the total samples were checked before and after data entry. Third, after data entry, the individual variable frequency was checked and finally, only the authorized person (researcher) handled the data.

### Data analysis

Data were cleaned, edited, and checked for accuracy and consistency. There was a categorical type of independent and dependent variables. The dependent variable was in a dichotomous character. Family and individual characters, socio-demographic characters, and IEC related variables were independent variables whereas enrolment of HI was the dependent variable. Some attributes of variables were lumped due to small frequency. Descriptive [frequencies and percentage] analysis for sample characters, bivariate [chi-square test] analysis to measure the association and measure the differences, and multivariate [logistic regression] analyses were performed to confirm the predictors. Variables having significant differences in bivariate analysis were further analysed and adjusted for multivariate analysis. Three models are presented in the multivariate analysis. Socio-demographic characteristics have been adjusted in model I, IEC related variables in model II, and finally all these variables are adjusted in model III. We used IBM SPSS Statistics 20 to analyse the data.

### Ethical consideration

NHRC reviewed and approved the study proposal on 15th February 2018. The National Ethical Guidelines for Health Research in Nepal and Standard Operating Procedure [28] and Ethical Compliance Checklist prepared by the American Psychological Association [29] were followed throughout the research process. All the respondents were informed about study objectives, time taken for interview, and right to reject at any time. Moreover, consent taken for participation voluntarily, established building rapport, repeated questions, and even translated in local languages as required.

### Potential biases and management

Households were randomly selected for enrolment to reduce selection bias. There was no discrimination among age, sex, and ethnicity. But the information was collected from household heads or senior members of the family assuming that they might have more information about their family, family members, access to IEC, and HI. The respondents were asked even crossed checked for some questions (having dichotomous character) to reduce the recall/response bias. Data collection was led by the researcher involving trained enumerators to reduce possible biases.

## Results

### Respondents’ characteristics

Of the total 810 respondents, 70% were from Kailali and 30% were from Baglung District. Out of them, more than one fourth [26%] were from rural areas. More than half of the respondents [51%] were female. Among them, two-third [66%] were the household heads. More than 92% were literate, more than half of them had a basic and secondary level of education, and 12% had a bachelor or higher level of education. Forty-one percent of households belonged to a nuclear family. Fifty-six percent of the total households had up to five members in the family, 42% of households had six to 10 members and nearly 2 % had more than 10 members in the family.

More than half [51%] of the total respondents could manage food for their family throughout the year from their own product; 16, 14 and 11% could manage their family food up to 3 months, 3 to 6 months, and 6 to 9 months respectively. Similarly, 8 % of them could manage food for their family for nine to 12 months with their own product. More than one third [34.6%] of the respondents expressed that minimum one family member had some type of chronic disease. Half of the respondents were enrolled and half others were not enrolled in the HIP which was already determined during sample size calculation and sample selection. Seventy-two percent of the participants expressed that they had some knowledge about health insurance and the remaining 28% did not have (Table 1).

**Table 1:** Background characteristics of households and participants

Variables	Category	Total (*n* = 810)	
		%	N
District	Baglung	30.1	244
	Kailali	69.9	566
Residence type	Urban	74.1	600
	Rural	25.9	210
Sex of respondents	Male	49.0	397
	Female	51.0	413
Household head	No	34.1	276
	Yes	65.9	534
Age group of respondents	Upto 20 years	2.8	23
	21 to 40 years	59.5	482
	41 to 60 years	28.8	233
	More than 60 years	8.9	72
Educational status	Illiterate	7.4	60
	Literate	30.4	246
	Basic education	26.4	214
	Secondary education	24.3	197
	Bachelor or above	11.5	93
Type of family	Nuclear	41.0	332
	Joint	59.0	478
Size of family	Up to 5 members	56.4	457
	6 to 10 members	42.0	340
	More than 10 members	1.6	13
Wealth status	Poor	33.3	270
	Middle	33.3	270
	Rich	33.3	270
Ability to feed the family throughout the year	No	48.8	395
	Yes	51.2	415
Family member having chronic diseases	No	65.4	530
	Yes	34.6	280
Enrolled in health insurance	No	50.0	405
	Yes	50.0	405
Ever heard about health insurance	No	28.0	227
	Yes	72.0	583
Sources of information^a^ (*n* = 583)	Neighbour/Peer	40.3	235
	Radio/FM	49.4	288
	Television	36.5	213
	Family members	14.8	86
	Health worker/Doctor	11.1	65
	Teacher	11.3	66
	FCHV	13.7	80
	Training/seminar	4.1	24
	Enrolment assistant	70.8	413
	Print media and others	3.8	22
Knowledge about the contribution amount	No	34.3	278
	Yes	65.7	532
Having HI related books	No	83.2	674
	Yes	16.8	136
Participated in HI related training	No	95.1	770
	Yes	4.9	40
Interaction with peers or neighbours about HI	No	68.0	551
	Yes	32.0	259
Known from social media	No	80.9	655
	Yes	19.1	155
Listened HI related information from Radio/FM	No	52.3	424
	Yes	47.7	386
Watched HI related information in TV	No	61.7	500
	Yes	38.3	310
Seen hoarding board	No	73.3	594
	Yes	26.7	216
Read newspaper	No	86.9	704
	Yes	13.1	106
Seen brochure/poster/pamphlet	No	82.5	668
	Yes	17.5	142

^a^ Multiple responses

Most of the participants [71%] were informed by the enrolment assistant. Nearly half [49%] of them were informed by radio/FM, 40% from neighbours/peers, 37% from TV, 15% from family members, 14% from female community health volunteers [FCHV], and 11% from teachers and health workers respectively. Nearly two-third [66%] of the respondents had information about the contribution amount for HI. Seventeen percent of them had HI related books or guidelines. However, only 5 % had participated in training and discussion related to HI.

Nearly one third [32%] of the participants had discussed with peers or neighbours about HI whereas 19% of them were informed through social media. Nearly half of the total respondents listened to HI related messages from radio whereas 38% of them watched HI related messages from TV. Data show that 27% of the respondents saw HI related messages on the hoarding board and 13% read HI related messages from newspapers. Eighteen percent of the respondents received HI related information from brochure, poster, pamphlet, and flyers.

### Family and respondents’ characteristics; information, education, and communication; and enrolment in health insurance

Out of the total respondents, 50% resided in the urban area and 49% of rural were enrolled. Fifty-three percent of the male respondents were enrolled in HI compared to 47% of females. Fifty-three percent of the respondents who were the household heads were enrolled in HI compared to 45% of those who were not household heads [*p* < 0.05]. Data show that the higher the age higher the enrolment rate. Twenty-six percent of the respondents of age less than 20 years were enrolled in HI compared to 44% from the age of 21 to 40 years, 59% from the age of 41 to 60 years, and 68% from the age of more than 60 years [*p* < 0.001]. There were no significant differences between the educational level of respondents, types of family, and size of the family; and enrolment in HI. More than half (56%) of respondents having rich wealth status were enrolled compared to 46% of middle and 49% poor wealth status [*p* < 0.05] (Table 2).

**Table 2:** Family characteristics and HI related information, and enrolment in HI

Variables	Category	Enrolled in health insurance					
		No		Yes		Chi-Square	*P*-Value
		N	%	N	%		
District	Baglung	122	50.0	122	50.0		
	Kailali	283	50.0	283	50.0		
Residence type	Urban	298	49.7	302	50.3	0.103	0.748
	Rural	107	51.0	103	49.0		
Sex of respondents	Male	186	46.9	211	53.1	3.088	0.079
	Female	219	53.0	194	47.0		
Household head	No	153	55.4	123	44.6	4.946	0.026
	Yes	252	47.2	282	52.8		
The age group of respondents	Upto 20 years	17	73.9	6	26.1	29.565	< 0.001
	21 to 40 years	270	56.0	212	44.0		
	41 to 60 years	95	40.8	138	59.2		
	More than 60 years	23	31.9	49	68.1		
Educational status	Illiterate	27	45.0	33	55.0	2.490	0.646
	Literate	132	53.7	114	46.3		
	Basic education	103	48.1	111	51.9		
	Secondary education	99	50.3	98	49.7		
	Bachelor or above	44	47.3	49	52.7		
Type of family	Nuclear	169	50.9	163	49.1	0.184	0.668
	Joint	236	49.4	242	50.6		
Size of family	Upto 5 members	231	50.5	226	49.5	0.935	0.626
	6 to 10 members	166	48.8	174	51.2		
	More than 10 members	8	61.5	5	38.5		
Wealth status	Poor	139	51.5	131	48.5	6.163	0.046
	Middle	147	54.4	123	45.6		
	Rich	119	44.1	151	55.9		
Ability to feed the family throughout the year	No	197	49.9	198	50.1	0.005	0.944
	Yes	208	50.1	207	49.9		
Family member having chronic diseases	No	292	55.1	238	44.9	15.913	< 0.001
	Yes	113	40.4	167	59.6		
Heard about health insurance	No	217	95.6	10	4.4	262.260	< 0.001
	Yes	188	32.2	395	67.8		
Sources of HI related information^a^ (*n* = 583)	Neighbour/Peer	74	31.5	161	68.5	102.328	< 0.001
	Radio/FM	101	35.1	187	64.9		
	Television	68	31.9	145	68.1		
	Family members	17	19.8	69	80.2		
	Health worker/Doctor	17	26.2	48	73.8		
	Teacher	17	25.8	49	74.2		
	FCHV	19	23.8	61	76.3		
	Training/seminar	6	25.0	18	75.0		
	Enrolment assistant	86	20.8	327	79.2		
	Print media and others	4	18.2	18	81.8		
Knowledge about the contribution amount	No	238	85.6	40	14.4	214.713	< 0.001
	Yes	167	31.4	365	68.6		
Having HI related books	No	382	56.7	292	43.3	71.577	< 0.001
	Yes	23	16.9	113	83.1		
Participated in HI related training	No	394	51.2	376	48.8	8.521	0.004
	Yes	11	27.5	29	72.5		
Interact with peers or neighbour about HI	No	335	60.8	216	39.2	80.376	< 0.001
	Yes	70	27.0	189	73.0		
Known from social media	No	336	51.3	319	48.7	2.306	0.129
	Yes	69	44.5	86	55.5		
Listened HI related information from Radio/FM	No	259	61.1	165	38.9	43.731	< 0.001
	Yes	146	37.8	240	62.2		
Watched HI related information in TV	No	290	58.0	210	42.0	33.445	< 0.001
	Yes	115	37.1	195	62.9		
Seen hoarding board	No	335	56.4	259	43.6	36.465	< 0.001
	Yes	70	32.4	146	67.6		
Read newspaper	No	367	52.1	337	47.9	9.769	0.002
	Yes	38	35.8	68	64.2		
Seen brochure or poster or pamphlet	No	359	53.7	309	46.3	21.348	< 0.001
	Yes	46	32.4	96	67.6		

Note: ^a^ = multiple responses

Sixty percent of the respondents, who had a family member(s) suffering from the chronic disease(s), those who were enrolled in HI compared to 45% who had not [*p*< 0.001]. Sixty-eight percent of the respondents who heard about HI were enrolled in HI compared to 4 % who did not [*p*< 0.001]. Eighty percent of the respondents, who got information from family members, were enrolled in HI compared to 79% from enrolment assistant, 76% from FCHV, 75% from training or seminars, 74% equally from teachers and health workers/doctors respectively, 69% from neighbours, 68% from TV, and 65% from Radio/FM [*p*< 0.001]. Sixty-nine percent of the respondents, who knew the contribution amount, were enrolled in HI compared to 14% who did not know the contribution amount [*p*< 0.001]. Eighty-three percent of the respondents, who had HI related books or guidelines, were enrolled in HI compared to 43% of those who had not HI related books or guidelines [*p*< 0.001].

Similarly, 73% of the respondents, who participated in training or discussion of HI related programme, were enrolled in HI compared to 9 % of those who did not participate [*p*< 0.01]. Seventy-three percent of the respondents, who discussed with peers or neighbours about HI related issues, were enrolled in HI compared to 39% of those who did not discuss [*p*< 0.001]. Sixty-two percent of the respondents, who listened to HI related information from Radio/FM, were enrolled in HI compare 39% of those who did not listen [*p*< 0.001]. Likewise, 63% of the respondents, who watched HI related messages from TV, were enrolled in HI compared to 42% of those who did not watch [*p*< 0.001]. Moreover, 68% of the respondents, who saw HI related messages from hoarding board [HB], were enrolled in HI compared to 44% of those who did not see HB [*p*< 0.001]. Sixty-four percent of the respondents, who read HI related messages from the newspaper, were enrolled in HI compared to 48% of those who did not read newspapers [*p*< 0.01]. Similarly, 68% of the respondents, who had seen HI related information from a brochure, poster, or pamphlet, were enrolled in HI compared to 46% of those who did not [*p*< 0.001].

### Multivariate analyses of background characteristics; exposure to IEC; and enrolment in HI

We used multivariate analysis in three models. In the first model, we included background characteristics and enrolment in HI. In the second model, we presented exposure to information, education, and communication; and enrolment in HI. Lastly, in the third model, all these variables were included/adjusted for further prediction. In the bivariate analysis, a chi-square test was used to test the association between the variables: socio-demographic characteristics; information, education and communication; and enrolment in HI. The variables were further examined (if significant in chi-square test) in the multivariate analysis in order to identify the significant predictors of the likelihood of enrolment in HI. During the process of analysis, multi-collinearity among the variables was assessed (Additional file 1). As none of the variables was highly correlated, all the variables were included in the logistic model.

According to Model I, it was found that the higher the age higher the chances of enrolment. Respondents age 21 to 40 years, 41 to 60 years, and more than 60 years were 1.9 [aOR=1.916] times, 3.2 times [aOR= 3.200, *p*< 0.05], and 4.4 times [aOR=4.352, p< 0.05] more likely to enrol in HI respectively compared to age less than 21 years but not significant in Model III. The model I shows, the respondents who had a family member(s) having a chronic disease(s) were more likely to enrol in HI [aOR = 1.536, *p*< 0.01] compared to the family who had no chronic disease(s) within family member(s) but the result was not consistent in Model III.

Interestingly, the model I and model III showed different results with regards to the sex of the respondents, household headship, wealth status, and chronic disease(s) within family member(s). The model I showed that females were more likely to enrol than males, while model III showed females were 41% more likely to enrol. In the same way, household headship, rich wealth status, and having chronic diseases were more likely to enrol but after adjusting all variables model III showed that these variables had lower odds ratios and were not statistically significant.

Model II shows the respondents who heard about health insurance were 20.5 times more likely to enrol compared to those who did not [aOR = 20.521, *p*< 0.001]. Similarly, the respondents who had knowledge about the contribution amount for health insurance were 4.9 times more likely to enrol than those who did not have [aOR = 4.925, *p*< 0.001]. Likewise, the respondents who had health insurance-related books or guidelines were 5.1 times more likely to enrol in HI than those who had not [aOR = 5.117, p< 0.001]. Interestingly, the respondents who interacted with peers or neighbours were 1.9 times more likely to enrol in HI compared to those who did not interact [aOR = 1.883, *p*< 0.01] (Table 3).

**Table 3:** Logistic regression of background characteristics, and exposure to communication; and enrolment in HI

		Model I			Model II			Model III		
Variables	Attributes		95% CI			95% CI			95% CI	
		aOR	Lower	Upper	aOR	Lower	Upper	aOR	Lower	Upper
*Sociodemographic characteristics*										
Sex^a^	Male (ref.)									
	Female	.977	.719	1.328				1.411	.929	2.142
Household head	No (ref.)									
	Yes	1.068	.763	1.494				.974	.623	1.521
Age group of respondents	Up to 20 years (ref.)									
	21 to 40 years	1.931	.729	5.119				1.377	.368	5.155
	41 to 60 years	3.212*	1.161	8.889				2.560	.874	10.179
	More than 60 years	4.353*	1.427	13.276				3.962	.874	17.950
Wealth status	Poor (ref.)									
	Middle	.820	.580	1.161				.586*	.359	.957
	Rich	1.201	.846	1.705				.627	.375	1.046
Family member having chronic diseases	No (ref.)									
	Yes	1.536**	1.130	2.090				.913	.610	1.365
*IEC related factors*										
Heard about health insurance	No (ref.)									
	Yes				20.521*****	10.020	42.025	21.183***	10.168	44.129
Knowledge about the contribution amount	No (ref.)									
	Yes				4.925***	3.049	7.953	5.128***	3.088	8.515
Have HI related books or guidelines	No (ref.)									
	Yes				5.117***	2.759	9.490	4.842***	2.610	8.981
Participated in HI related training	No (ref.)									
	Yes				.428	.179	1.023	.426	.175	1.036
Interact with peers and neighbours about HI	No (ref.)									
	Yes				1.883**	1.244	2.851	1.736**	1.139	2.646
Listened HI related info from Radio/FM	No (ref.)									
	Yes				.917	.611	1.375	.941	.622	1.422
Watched HI related information in TV	No (ref.)									
	Yes				.831	.551	1.255	.940	.607	1.455
Seen HI related hoarding board	No (ref.)									
	Yes				1.342	.827	2.178	1.473	.899	2.413
Read HI related newspaper	No (ref.)									
	Yes				.653	.359	1.188	.731	.398	1.342
Seen HI related brochure/poster/pamphlet	No (ref.)									
	Yes				.669	.375	1.194	.709	.394	1.276
Area Under Curve (AUC)		62.8%			85.5%			86.6%		

Note: *significant at *p*< 0.05, ** significant at *p*< 0.01, ***significant at *p*< 0.001. *aOR* Adjusted odds ratio. ^a^ = variable not significant in bivariate analysis but included in multivariate analysis

Model III shows some similar and some contradictory projection compared to model I and model II. Age groups more than 20 years were more likely to enrol in HI compared to age up to 20 years which was the similar prediction with model I. Model II and model III have nearly the same result compared to model I. The respondents who heard about HI were more likely to enrol in HI [aOR = 20.229, *p*< 0.001] compared to those who did not. The result seems similar to model II. In the same way, the respondents who had heard about HI, knowledge on contribution amount, were more likely to enrol in HI [aOR = 5.176, *p*< 0.001] compared to those who had not. The respondents having HI related books or guidelines were more likely to enrol in HI [aOR = 4.812, p< 0.001] that was also the same result with model II. The participants who had participated in the HI related training were less likely to enrol in HI compared to those who did not.

Interaction with peers and neighbours played a positive role in enrolment. The respondents who interacted about HI with neighbours or peers were 1.7 times more likely to enrol in HI [aOR = 1.739, *p*< 0.01] compared to those who did not. The result was the same as model II. Multivariate analysis shows that radio and/or TV had no more influencing role in enrolment. Similarly, newspaper, poster, pamphlet, flyer, or brochure had no positive and influencing role to the enrolment in HI. But The participants who saw HI related messages from the hoarding board were 1.3 times more likely to enrol in HI compared to those who did not.

## Discussion

### Key results and interpretations

IEC contains different approaches, activities, and methods that are targeted to change the desirable behavior through the application of various activities by creating awareness, upgrading knowledge, changing desirable attitude, and supporting individuals for adopting innovation or desirable behavior [14, 30–32]. In this study, interaction and discussion with peers or neighbours seemed to contribute more to HI related communication. IEC materials were useful tools for promoting suitable eye awareness and also powers for social change in Madurai, India [33]. Similarly, it was observed that IEC and contraceptive uses were significantly associated beyond the visits of medical and family planning officers which was experienced in Indonesia [34]. A study from Gambia shows that mass media was an effective and feasible means to make a change in maternal health service utilization and care [35]. A similar observation seemed in India that IEC approaches appeared appropriate for consuming a low salt diet to control hypertension [36] however, flip chart seemed ineffective for food hygiene and food safety [37]. IEC could be useful not only for making changes in behaviour but also for preparedness, response, and mitigation for disaster that may save lives and resources [38].

Different audiences may be motivated by a different mode of communication. Arroz (2017) states that radio, dramas, lectures, posters and pamphlets, and folk programmes could be considered as synergetic approaches but not conceal one another [39]. There was a significant difference between the respondents who listened to HI messages from radio and enrolment in HI compared to those who did not. A similar result was observed in Liberia that the women who listened to radio spots were encouraged to care for their child and visit health facilities of their babies to appear with fever [40].

The study shows that the educational level of respondents was not significantly associated with the enrolment but heard about HI was significantly associated with the enrolment in HI. A study from Nigeria shows that the educational level of the participants was significantly associated with the awareness of the national health insurance scheme [41]. So it does not always mean that educational status is equal to HI literacy as well as enrolment. Another study from Columbia suggested that integrated approaches (radio, TV, and interpersonal communication with health workers/volunteers) were effective for seeking treatment for malaria [42]. Consequently, a study from Odisha, India shows that drug adherence to IEC was significantly higher in receiving Artemisia in combination therapy in the experimental group compared to control [43]. Therefore, it can be concluded that IEC is an effective means to adopt an innovation or change in the desired behaviour.

The study shows that nearly two-thirds of the respondents, who interacted with peers or neighbours were enrolled compared to those who did not interact that was statistically significant. In the same way, interaction with peers or neighbours was a positive significant predictor for enrolment in HI. Various empirical studies support the argument that information and counselling from neighbours or peers make significant changes in behaviour modification. Not only good behaviour but also health destructive behaviours influenced by peers [44]. Peer teaching or coaching enhances relationship, reciprocal understanding, and development to achieve the targeted behaviour [45]. Besides these, peer assessment improves students’ learning outcomes with progressive attitudes [46]. Not only that, but the peering approach appears also successful in peer to peer fiscal planning and educational programmes [47].

The peer teaching method supports the development of in-depth and mutual understanding, cooperative and collaborative learning environment, and also ensures self-assessment and monitoring of progress [48]. The peering approach seems more effective especially for adolescents with a high-risk background. It connects with positive towards peer-to-peer relationships and they should be guided in supporting one-another in promoting healthy behaviour [49]. The approach has been recognized as an effective and valuable approach so it can be incorporated into different settings using various methods and approaches [50] which might be fastest, cheapest, efficient, and beneficial and can be utilized social as well as a cognitive field [51].

The peering or neighbouring approach leads to productive social interaction, responsiveness, co-operation, and positive attitudes, and social harmony. It supports the learning environment and encourage participation in an interaction [52]. A systematic review shows that adolescents and sexual health education had improved in knowledge, attitude and intentions by peer leading approach [53]. Peer mediated approach also leads to positive changes in the social behaviour of a person having learning disabilities [54]. Another experimental study shows that peer education significantly increases the knowledge and practice of the mental health of adolescents girls [55].

Peers/neighbours can support in three different ways: first, social; second, informational; and lastly, personal or folk, facts, and feelings respectively which are interconnected with interpersonal skills. From the biomedical point of view on breastfeeding, peer to peer [P2P] approach is women-centred, related to their own experiences, considering women as a change agent from their own experiences and able to cope with cultural constraints, therefore, recommended for P2P approach [56]. The result of this study and empirical evidence from other studies show that P2P or neighbouring approach is a more convenient, efficient and effective way to change or modify the behaviour.

## Limitations

The study was conducted in Baglung and Kailali Districts since the HIP was initially implemented in these districts which could limit the ability to generalize the results throughout the nation. The article has mainly focused on IEC activities and assessing their likely association with enrolment in HI. Consequently, the sample size was taken equally from enrolled and non-enrolled households assuming that they have equal access to IEC. Since all selected variables were measured at a single point of time, the results can only predict a particular time context. It might be a potential bias in the study. Moreover, a cross-sectional study could not show the cause-effect relationship. Some variables were missed in the data such as household’s cash income; quality health services provided by the health facilities; social media use like Facebook, YouTube; and households’ satisfaction which may influence the enrolment. A mixed-method study can be conducted covering wider areas addressing the limitation as mentioned above in future studies.

## Conclusion

From the data of the study and empirical evidence from other studies, it can be concluded that hearing about HI and knowledge about contribution amount seems to be a predictor of enrolment. Similarly, HI related books, guidelines, and hoarding board can support mass participation. The existing ways of message dissemination through radio, TV, newspaper, poster, and pamphlet seem less effective for enrolment. It would be better to be re-evaluated for disseminating message to public awareness or it could be modified for betterment. But, interaction with peers or neighbours seemed a positive and significant predictor for enrolment in HI. Therefore, it should be taken into account while planning IEC interventions.

## Supplementary Information

**Additional file 1.**

## Data Availability

The datasets used and/or analysed during the current study are available from the corresponding author on reasonable request.

## Abbreviations

aOR: Adjusted Odds Ratio
CoN: Constitution of Nepal
GoN: Government of Nepal
HH: Household Head
HI: Health Insurance
HIA: Health Insurance Act
HIB: Health Insurance Board
HIP: Health Insurance Programme
IEC: Information, education, and Communication
IS: Interview Schedule
NDHS: Nepal Demographic and Health Survey
NHRC: Nepal Health Research Council
SDGs: Sustainable Development Goals
SHS: Social Health Security
SPSS: Statistical Package for Social Sciences
UHC: Universal Health Coverage

## References

[1] National Planning Commission. Sustainable Development Goals 2016–2030: National {Prelinimary} Report. Singha Durbar: National Planning Commission; 2015. https://www.undp.org/content/dam/nepal/docs/reports/SDGfinalreport-nepal.pdf.

[2] Nepal Law Commission (2015). The Constitution of Nepal.

[3] Department of Health Services. Annual Report: Department of Health Services 2074/75 (2017/18). Kathmandu: Department of Health Services; 2019. https://dohs.gov.np/wp-content/uploads/2019/07/Annual_Report_2074-751.pdf.

[4] Member of Parliament Nepal (2017). Health Insurance Act, 2074.

[5] Health Insurance Board. Annual Report FY 2073/74(2016/17). Kathmandu: Health Insurance Board; 2017. https://hib.gov.np/public/uploads/shares/downloads/Annual_Report_of_SHSP_2073_74_Nepali.pdf.

[6] KOICA-Nepal Health Insurance Support Project [NHISP]. Comprehensive district assessment for health insurance in Kailali district. Lalitpur: KOICA-Nepal Health Insurance Support Project [NHISP]; 2014.

[7] Health Insurance Board. Annual Report: Fiscal Year 2074/75. Kathmandu: Health Insurance Board; 2019. https://hib.gov.np/public/uploads/shares/hib_nepal_annual_report_2075_complete.pdf.

[8] Acharya D, Devkota B, Adhikari R (2018). Willingness to pay for family health insurance : evidence from Baglung and Kailali districts of Nepal. Glob J Health Sci.

[9] KOICA/HIMAL Project (2012). Community-based health insurance: Implementation manual.

[10] Ghimire R (2012). Community based health insurance practices in Nepal.

[11] Social Health Security Development Committee. Communication strategy on social health security in Nepal. Kathmandu: Social Health Security Development Committee; 2015.

[12] Mefalopulos P (2008). Development communication sourcebook : broadening the boundaries of communication.

[13] Mundorf N, Redding CA, Paiva AL (2018). Sustainable transportation attitudes and health behavior change: Evaluation of a brief stage-targeted video intervention. Int J Environ Res Public Health.

[14] World Health Organization (2001). Information, education, and communication: Lessons from the past; perspectives for the future.

[15] Zimbabwe National Family Planning Council (1998). IEC reference manual for health programme managers.

[16] Piotrow PT, Kincaid DL, Rimon JGI, Rinehart W (1997). Health communication: lessons from family planning and reproductive health.

[17] Ministry of Health, New ERA, ICF (2017). Nepal Demographic and Health Survey 2016.

[18] Water Aid. Behaviour Change Theory -Outline [Module I] (n.d., pp 1–12). https://www.lshtm.ac.uk/media/11191.

[19] Barker K (2003). Order from chaos: organizational aspects of information, education, and communication (a case study from Mali). J Health Commun.

[20] Iordache-Platis M, Josan I (2009). Communication efficiency within higher education institutions : the case of Romania. Eur Res Stud.

[21] Puri S (2017). The role of information, education and communication (IEC) in sustainable solid waste management. Open Access Int J Sci Eng.

[22] Ubiquitous Information Society Advisory Board (2010). National plan for educational use of information and communications technology.

[23] Sample size calculator [Internet]. SurveyMonkey. 2019. Available from: https://www.surveymonkey.com/mp/sample-size-calculator/. Accessed 5 Jan 2019.

[24] Central Bureau of Statistics. Statistical pocket book of Nepal. Kathmandu: Central Bureau of Statistics; 2014.

[25] Acharya D, Devkota B, Wagle BP (2019). Factors associated to the enrollment in health insurance: an experience from selected districts of Nepal. Asian Soc Sci.

[26] Naing L, Winn T, Rusli BN (2006). Practical issues in calculating the sample size for prevalence studies. Arch Orofac Sci.

[27] Hair JF, Black WC, Babin BJ, Anderson RE (2014). Multivariate Data Analysis.

[28] Nepal Health Research Council (2011). National ethical guidelines for health research in Nepal and standard operating procedures.

[29] American Psychological Association (2010). Publication manual of the American Psychological Association.

[30] Akhund S, Avan BI (2011). Development and pretesting of an information , education and communication ( IEC ) focused antenatal care handbook in Pakistan. BMC Res Notes.

[31] World Health Organization. WHO/UNICEF regional workshop on information, education and communication on health. Manila: World Health Organization; 1981. https://apps.who.int/iris/bitstream/handle/10665/207248/ICP_INF_001_eng.pdf?sequence=1&isAllowed=y.

[32] World Health Organization. Information, education and communication: A guide for AIDS programme managers. New Delhi: World Health Organization; 2000. https://apps.who.int/iris/bitstream/handle/10665/205344/B0224.pdf?sequence=1&isAllowed=y.

[33] Abraham Pradeep G, Sheeladevi S. Lions Aravind Institute of Community Ophthalmology. Madura; Vision 2020 e-resource. http://v2020eresource.org/content/files/IEC_article.pdf.

[34] Winarni E, Dawam M (2016). Family planning information, education and communication with contraceptive use. Natl Public Heal J.

[35] Anya SE, Hydara A, Jaiteh LES (2008). Antenatal care in the Gambia: missed opportunity for information, education and communication. BMC Pregnancy Childbirth.

[36] Borah PK, Kalita HC, Paine SK, Khaund P, Bhattacharjee C, Hazarika D (2018). An information, education and communication module to reduce dietary salt intake and blood pressure among tea garden workers of Assam. Indian Heart J.

[37] Takanashi K, Quyen DT, Hoa NTL, Khan NC, Yasuoka J, Jimba M (2013). Long-term impact of community-based information, education and communication activities on food hygiene and food safety behaviors in Vietnam : A longitudinal study. PLoS One.

[38] Crane O, Balen J, Devkota B, Ghimire S, Rushton S (2017). Use of information and communication technologies in the formal and informal health system responses to the 2015 Nepal earthquakes. Health Policy Plan.

[39] Arroz JAH (2017). Social and behavior change communication in the fight against malaria in Mozambique. Rev Saude Publica.

[40] Awantang G, Babalola S, Koenker H, Fox K, Toso M, Lewicky N (2018). Correlates of social behavior change communication on care - seeking behaviors for children with fever : an analysis of malaria household survey data from Liberia. Malar J.

[41] Adewole DA, Akanbi SA, Osungbade KO, Bello S (2017). Expanding health insurance scheme in the informal sector in Nigeria: awareness as a potential demand-side tool. Pan Afr Med J.

[42] Canavati SE, De Beyl CZ, Ly P, Shafique M, Boukheng T, Rang C (2016). Evaluation of intensified behaviour change communication strategies in an artemisinin resistance setting. Malar J.

[43] Swain TR, Raulo A, Mohapatra N, Singha MR (2015). Information education and communication can improve adherence to Artemether-Lumefantrine combination in patients of uncomplicated falciparum malaria. J Clin Diagn Res.

[44] de Vries H, Candel M, Engels R, Mercken L (2006). Challenges to the peer influence paradigm: results for 12–13 year olds from six European countries from the European smoking prevention framework approach study. Tob Control.

[45] Parker P, Wasserman I, Kram KE, Hall DT (2015). A relational communication approach to peer coaching. J Appl Behav Sci.

[46] Friedman BA, Cox PL, Maher LE (2008). An expectancy theory motivation approach to peer assessment. J Manag Educ.

[47] Goetz JW, Durband DB, Halley R, Davis K (2011). A peer-based financial planning and education service program : an innovative pedagogic approach a peer-based financial planning. J Coll Teach Learn.

[48] Ramaswamy S, Harris I, Tschirner U (2001). Student peer teaching : an innovative approach to instruction in science and engineering education. J Sci Educ Technol.

[49] Eggert LL, Nicholas LJ, Owen LM (1995). Reconnecting youth: a peer group approach to building life skills.

[50] Evans DJR, Cuffe T (2009). Near-peer teaching in anatomy : an approach for deeper learning. Anat Sci Educ.

[51] Coenen ME (2002). Using gifted students as peer tutors: an effective and beneficial approach. Winter..

[52] Hwang G, Chang S (2016). Effects of a peer competition-based mobile learning approach on students’ affective domain exhibition in social studies courses. Br J Educ Technol.

[53] Ranabhat CL, Kim C, Singh A, Acharya D, Pathak K (2019). Challenges and opportunities towards the road of universal health coverage ( UHC ) in Nepal: a systematic review. Arch Public Heal.

[54] Moore T, Carey L (2005). Friendship formation in adults with learning disabilities : peer- mediated approaches to social skills development. Br J Learn Disabil.

[55] Taghdist MH, Noori SM, Merghati KE, Hoseini F, Asgharnejad FAA (2012). Impact peer education approach on knowledge and practice about mental health of adolescent girls. Toloo-E-Behdasht.

[56] Dykes F. Training breastfeeding peer supporters: an enabling approach. Matern Child Nutr. 2005;1(1):59–60.

