# Supplementary material for: Association of information, education, and communication with enrolment in health insurance: a case of Nepal
Source: Arch Public Health. 2020 Dec 14;78:135. doi: 10.1186/s13690-020-00518-8 (PMC7737309; doi:10.1186/s13690-020-00518-8)
Supplement: Supplementary file 1 — Additional file 1. [file 13690_2020_518_MOESM1_ESM.docx]

**Appendix I**

Multi-collinearity check among the variables

| **Correlations [among socio-demographic characteristics]** | | | | | | |
| --- | --- | --- | --- | --- | --- | --- |
|  | | Sex of respondents | Household head | Age group of respondents | Wealth status | Family member having chronic diseases |
| Sex of respondents | Pearson Correlation | 1 | -.309^**^ | -.280^**^ | -.085^*^ | -.051 |
|  | Sig. (2-tailed) |  | .000 | .000 | .016 | .149 |
|  | N | 810 | 810 | 810 | 810 | 810 |
| Household head | Pearson Correlation | -.309^**^ | 1 | .371^**^ | -.032 | .030 |
|  | Sig. (2-tailed) | .000 |  | .000 | .365 | .400 |
|  | N | 810 | 810 | 810 | 810 | 810 |
| Age group of respondents | Pearson Correlation | -.280^**^ | .371^**^ | 1 | .127^**^ | .261^**^ |
|  | Sig. (2-tailed) | .000 | .000 |  | .000 | .000 |
|  | N | 810 | 810 | 810 | 810 | 810 |
| Wealth status | Pearson Correlation | -.085^*^ | -.032 | .127^**^ | 1 | .064 |
|  | Sig. (2-tailed) | .016 | .365 | .000 |  | .070 |
|  | N | 810 | 810 | 810 | 810 | 810 |
| Family member having chronic diseases | Pearson Correlation | -.051 | .030 | .261^**^ | .064 | 1 |
|  | Sig. (2-tailed) | .149 | .400 | .000 | .070 |  |
|  | N | 810 | 810 | 810 | 810 | 810 |
| **. Correlation is significant at the 0.01 level (2-tailed). | | | | | | |
| *. Correlation is significant at the 0.05 level (2-tailed). | | | | | | |

**Correlations [among information, education and communication]**

|  | | | Heard about health insurance | Knowledge about contribution amount | Have HI related books | Participated in HI related training/workshop | Interact with peer or neighbour about HI | Listened HI related information from Radio/FM | | Watched HI related information in TV | Seen hoarding board | Read newspaper | | Seen brochure/poster/pamphlet |
| --- | --- | --- | --- | --- | --- | --- | --- | --- | --- | --- | --- | --- | --- | --- |
| Heard about health insurance | | Pearson Correlation | 1 | .603^**^ | .214^**^ | .142^**^ | .339^**^ | .359^**^ | | .271^**^ | .258^**^ | .193^**^ | | .230^**^ |
|  |  | Sig. (2-tailed) |  | .000 | .000 | .000 | .000 | .000 | | .000 | .000 | .000 | | .000 |
|  |  | N | 810 | 810 | 810 | 810 | 810 | 810 | | 810 | 810 | 810 | | 810 |
| Knowledge about contribution amount | | Pearson Correlation | .603^**^ | 1 | .199^**^ | .129^**^ | .295^**^ | .346^**^ | | .355^**^ | .295^**^ | .188^**^ | | .224^**^ |
|  |  | Sig. (2-tailed) | .000 |  | .000 | .000 | .000 | .000 | | .000 | .000 | .000 | | .000 |
|  |  | N | 810 | 810 | 810 | 810 | 810 | 810 | | 810 | 810 | 810 | | 810 |
| Have HI related books | | Pearson Correlation | .214^**^ | .199^**^ | 1 | .355^**^ | .273^**^ | .134^**^ | | .190^**^ | .230^**^ | .266^**^ | | .349^**^ |
|  |  | Sig. (2-tailed) | .000 | .000 |  | .000 | .000 | .000 | | .000 | .000 | .000 | | .000 |
|  |  | N | 810 | 810 | 810 | 810 | 810 | 810 | | 810 | 810 | 810 | | 810 |
| Participated in HI related training/workshop | | Pearson Correlation | .142^**^ | .129^**^ | .355^**^ | 1 | .259^**^ | .136^**^ | | .161^**^ | .249^**^ | .283^**^ | | .315^**^ |
|  |  | Sig. (2-tailed) | .000 | .000 | .000 |  | .000 | .000 | | .000 | .000 | .000 | | .000 |
|  |  | N | 810 | 810 | 810 | 810 | 810 | 810 | | 810 | 810 | 810 | | 810 |
| Interact with peer or neighbour about HI | | Pearson Correlation | .339^**^ | .295^**^ | .273^**^ | .259^**^ | 1 | .305^**^ | | .283^**^ | .293^**^ | .236^**^ | | .331^**^ |
|  |  | Sig. (2-tailed) | .000 | .000 | .000 | .000 |  | .000 | | .000 | .000 | .000 | | .000 |
|  |  | N | 810 | 810 | 810 | 810 | 810 | 810 | | 810 | 810 | 810 | | 810 |
| Listened HI related information from Radio/FM | | Pearson Correlation | .359^**^ | .346^**^ | .134^**^ | .136^**^ | .305^**^ | 1 | | .332^**^ | .325^**^ | .319^**^ | | .314^**^ |
|  |  | Sig. (2-tailed) | .000 | .000 | .000 | .000 | .000 |  | | .000 | .000 | .000 | | .000 |
|  |  | N | 810 | 810 | 810 | 810 | 810 | 810 | | 810 | 810 | 810 | | 810 |
| Watched HI related information in TV | | Pearson Correlation | .271^**^ | .355^**^ | .190^**^ | .161^**^ | .283^**^ | .332^**^ | | 1 | .289^**^ | .327^**^ | | .231^**^ |
|  |  | Sig. (2-tailed) | .000 | .000 | .000 | .000 | .000 | .000 | |  | .000 | .000 | | .000 |
|  |  | N | 810 | 810 | 810 | 810 | 810 | 810 | | 810 | 810 | 810 | | 810 |
| Seen hoarding board | | Pearson Correlation | .258^**^ | .295^**^ | .230^**^ | .249^**^ | .293^**^ | .325^**^ | | .289^**^ | 1 | .445^**^ | | .530^**^ |
|  |  | Sig. (2-tailed) | .000 | .000 | .000 | .000 | .000 | .000 | | .000 |  | .000 | | .000 |
|  |  | N | 810 | 810 | 810 | 810 | 810 | 810 | | 810 | 810 | 810 | | 810 |
| Read newspaper | | Pearson Correlation | .193^**^ | .188^**^ | .266^**^ | .283^**^ | .236^**^ | .319^**^ | | .327^**^ | .445^**^ | 1 | | .505^**^ |
|  |  | Sig. (2-tailed) | .000 | .000 | .000 | .000 | .000 | .000 | | .000 | .000 |  | | .000 |
|  |  | N | 810 | 810 | 810 | 810 | 810 | 810 | | 810 | 810 | 810 | | 810 |
| Seen brochure/poster/pamphlet | | Pearson Correlation | .230^**^ | .224^**^ | .349^**^ | .315^**^ | .331^**^ | .314^**^ | | .231^**^ | .530^**^ | .505^**^ | | 1 |
|  |  | Sig. (2-tailed) | .000 | .000 | .000 | .000 | .000 | .000 | | .000 | .000 | .000 | |  |
|  |  | N | 810 | 810 | 810 | 810 | 810 | 810 | | 810 | 810 | 810 | | 810 |
| **. Correlation is significant at the 0.01 level (2-tailed). | | | | | | | | | | | | | | |
| **Coefficients^a^** | | | | | | | | | | | | | | |
| Model | | | | | | | | | Collinearity Statistics | | | | | |
|  |  |  |  |  |  |  |  |  | Tolerance | | | | VIF | |
| I | Sex of respondents | | | | | | | | .868 | | | | 1.152 | |
|  | Household head | | | | | | | | .805 | | | | 1.242 | |
|  | Age group of respondents | | | | | | | | .762 | | | | 1.312 | |
|  | Wealth status | | | | | | | | .971 | | | | 1.030 | |
|  | Family member having chronic diseases | | | | | | | | .926 | | | | 1.080 | |
| a. Dependent Variable: Enrolled in health insurance | | | | | | | | | | | | | | |
| **Coefficients^a^** | | | | | | | | | | | | | | |
| Model | | | | | | | | | Collinearity Statistics | | | | | |
|  |  |  |  |  |  |  |  |  | Tolerance | | | | VIF | |
| II | Heard about health insurance | | | | | | | | .589 | | | | 1.697 | |
|  | H about contribution amount | | | | | | | | .579 | | | | 1.728 | |
|  | Have HI related books | | | | | | | | .780 | | | | 1.282 | |
|  | Participated in HI related training/workshop | | | | | | | | .804 | | | | 1.244 | |
|  | Interact with peer or neighbour about HI | | | | | | | | .754 | | | | 1.326 | |
|  | Listened HI related information from Radio/FM | | | | | | | | .734 | | | | 1.363 | |
|  | Watched HI related information in TV | | | | | | | | .761 | | | | 1.314 | |
|  | Seen hoarding board | | | | | | | | .635 | | | | 1.575 | |
|  | Read newspaper | | | | | | | | .649 | | | | 1.542 | |
|  | Seen brochure/poster/pamphlet | | | | | | | | .573 | | | | 1.744 | |
| a. Dependent Variable: Enrolled in health insurance | | | | | | | | | | | | | | |

| **Coefficients^a^** | | | |
| --- | --- | --- | --- |
| Model | | Collinearity Statistics | |
|  |  | Tolerance | VIF |
| III | Sex of respondents | .833 | 1.201 |
|  | Household head | .789 | 1.267 |
|  | Age group of respondents | .739 | 1.353 |
|  | Wealth status | .815 | 1.227 |
|  | Family member having chronic diseases | .896 | 1.116 |
|  | Heard about health insurance | .582 | 1.717 |
|  | Knowledge about contribution amount | .560 | 1.786 |
|  | Have HI related books | .776 | 1.288 |
|  | Participated in HI related training/workshop | .791 | 1.264 |
|  | Interact with peer or neighbour about HI | .743 | 1.347 |
|  | Listened HI related information from Radio/FM | .728 | 1.373 |
|  | Watched HI related information in TV | .694 | 1.441 |
|  | Seen hoarding board | .624 | 1.602 |
|  | Read newspaper | .644 | 1.554 |
|  | Seen brochure/poster/pamphlet | .569 | 1.756 |
| a. Dependent Variable: Enrolled in health insurance | | | |
